# Patient’s perspective on early treatment retention in take home buprenorphine maintenance treatment- an explorative study from India

**DOI:** 10.1192/j.eurpsy.2021.1511

**Published:** 2021-08-13

**Authors:** P. M, P. Chand, P. Murthy

**Affiliations:** 1 Psychiatry, AARUPADAI VEEDU MEDICAL COLLEGE AND HOSPITAL, PUDUCHERRY, India; 2 Psychiatry, NATIONAL INSTITUTE OF MENTAL HEALTH AND NEURO SCIENCES, BANGALORE, India

**Keywords:** opioid, qualitative, addiction, buprenorphine

## Abstract

**Introduction:**

Currently, Buprenorphine maintenance therapy (BMT) is an evidence-based treatment in retaining patients who are dependent on opioids. However, factors influencing retention are often measured objectively. Studies on patient’s perspectives on take home BMT in developing countries are limited.

**Objectives:**

This study examines the potential factors influencing treatment compliance in the early phase of Buprenorphine maintenance treatment from the patient’s perspective

**Methods:**

Participants (n=89) who were initiated on BMT were recruited and followed after six weeks. A semi-structured interview was conducted with 62 patients who remained in treatment and 24 patients who dropped out of the study

**Results:**

Based on the semi qualitative analysis some of the factors which facilitated the patient’s retention in treatment were: (1) Effectiveness in blocking withdrawal symptoms (2) effectiveness in reducing their cravings and controlling their opioid use (3) decreased fear of withdrawal and/or missing doses(4) improvement in the quality of life(5) patient-related factors like family support (6) effectiveness of the treatment program. Around nine percent of patients reported family support as the reason for retention, which is not noticed in other studies. Barriers reported by the patients while on medication were: (1) negative effect experienced with medication (2) program related difficulties like distance, unavailability (3) major life event interrupting the treatment (4) patient-related factors like low mood, financial constraints.

**Conclusions:**

Understanding factors associated with barriers to treatment provide insights into preventable factors that contribute to premature drop out from BMT and to improve clinical practice, policy decisions, or future research.

